# Epigenetic Therapy in Lung Cancer

**DOI:** 10.3389/fonc.2013.00135

**Published:** 2013-05-30

**Authors:** Stephen V. Liu, Muller Fabbri, Barbara J. Gitlitz, Ite A. Laird-Offringa

**Affiliations:** Norris Comprehensive Cancer Center, Keck School of Medicine, University of Southern California, Los Angeles, CA, USA

**Keywords:** epigenetics, DNA methylation, hypomethylating agent, histone deacetylase inhibitors, microRNAs, lung cancer

## Abstract

Epigenetic deregulation of gene function has been strongly implicated in carcinogenesis and is one of the mechanisms contributing to the development of lung cancer. The inherent reversibility of epigenetic alterations makes them viable therapeutic targets. Here, we review the therapeutic implications of epigenetic changes in lung cancer, and recent advances in therapeutic strategies targeting DNA methylation and histone acetylation.

## Introduction

Recent conceptual and technological advances have expanded our collective understanding of epigenetics and its role in the development and, potentially, the treatment of cancer. Epigenetics broadly refers to the study of alterations in gene expression caused by mechanisms other than direct changes in DNA sequence. Epigenetic processes include DNA methylation, histone modifications, chromatin organization, microRNAs (miRNAs), and other non-coding RNAs, and the cellular complement of transcription factors and other gene-regulatory DNA-binding proteins. Because epigenetic properties are retained during cell division, they play a key role in determining and maintaining cell phenotype. The most developed areas of epigenetics are the study of DNA methylation, histone modifications, and miRNA expression. Deregulation of epigenetic processes has been strongly implicated in all types of cancer including lung cancer, causing silencing of tumor suppressor genes, and activation of tumor promoting genes (Baylin and Jones, 2011). In addition, the interplay between epigenetic and genetic deregulation can contribute to cancer development and progression, for example, through the mutation of epigenetic regulator genes or the epigenetic deregulation of genes involved in DNA repair (Shen and Laird, 2013). Epigenetic changes have also been associated with resistance to traditional cytotoxic therapy (Sharma et al., 2010).

Epigenetic modifications are potentially reversible, and are therefore being aggressively pursued as therapeutic targets. This strategy has proven successful in several types of malignancy; decitabine, and 5-azacytidine are hypomethylating agents approved for the treatment of myelodysplastic syndromes (Daskalakis et al., 2002; Yang et al., 2010) and romidepsin is a histone deacetylase (HDAC) inhibitor approved in peripheral T-cell lymphoma (Coiffier et al., 2012). Currently, epigenetic therapies are being investigated for the treatment of many solid tumor types. Here, we review some of the recent advances in epigenetic therapy for lung cancer. Due to space limitations, cited references on general concepts will largely be restricted to review manuscripts.

## DNA Methylation

DNA methylation consists of the covalent addition of a methyl group to a cytosine residue in the context of a cytosine-phosphate-guanine (CpG) dinucleotide (Jones, 2012). Regions of DNA with a high frequency of CpG dinucleotides are termed CpG islands, and are found in the promoters of about 40% of human genes. Methylation of these CpG islands by DNA methyltransferases (DNMTs) can lead to gene silencing. It has become clear that methylation of specific promoters and the resulting impact on transcription play a pivotal role in carcinogenesis (Laird, 2005; Kanai and Hirohashi, 2007). The association between promoter methylation and lung cancer has been widely explored. A classic example is the silencing of cell cycle control gene *p16/INK4a*, first described in 1995 (Merlo et al., 1995). Methylation of *p16* was also assayed in surgical lung cancer specimens and detected at high rates in tumor tissue compared to normal lung (Kim et al., 2001; Zöchbauer-Muller et al., 2001). Interestingly, promoter methylation for *p16* has been associated with tobacco use (Belinsky et al., 1998; Belinsky et al., 2002; Yanagawa et al., 2002), the greatest risk factor for developing lung cancer. Methylation of *p16* and many other genes in lung cancer is now widely documented and genome-wide profiling has revealed subgroups of tumors with specific patterns (Tsou et al., 2007; Anglim et al., 2008; Carvalho et al., 2012; Kwon et al., 2012; Lockwood et al., 2012; Selamat et al., 2012; Shinjo et al., 2012; Walter et al., 2012; Wilkerson et al., 2012; Heller et al., 2013; Park et al., 2013).

Such specific DNA methylation patterns can provide useful information not only about the molecular basis of lung cancer development, but also for patient prognosis. The investigation of this goldmine of epigenetic data has only just begun. Early candidate gene studies using a nested case-control analysis of patients with stage I non-small cell lung cancer (NSCLC), showed that the methylation status of four genes (*p16, CDH13, RASSF1A*, and *APC*) correlated with recurrence (Brock et al., 2008). Importantly, methylation profiles may be equally useful as predictive markers. It was recently shown that the DNA methylation pattern in NSCLC cell lines and tissue samples correlates with phenotypic subsets associated with sensitivity to epidermal growth factor receptor (EGFR) inhibitors (Walter et al., 2012). Similarly, the methylation of *IGFBP-3* has been correlated with cisplatin resistance in NSCLC specimens (Ibanez de Caceres et al., 2010).

Besides its promise as a prognostic and predictive biomarker, DNA methylation has also emerged as a promising therapeutic target in lung cancer, primarily through the use of DNMT inhibitors. 5-azacytidine is a DNMT inhibitor activated by phosphorylation and incorporated into DNA and RNA. Because the enzyme is unable to methylate the base, it becomes trapped on the DNA and is targeted for proteasomal degradation, globally reducing DNA methylation levels (Jones et al., 1983; Schermelleh et al., 2005). In model systems, 5-azacytidine demonstrates intriguing antitumor activity. For example, in the H1299 lung cancer cell line, several genes are silenced by methylation, including *p16*, *FHIT*, and *WWOX*. When these cells were then implanted into nude mice, treatment with 5-azacytidine restored expression of these proteins and suppressed tumor growth (Cantor et al., 2006). Decitabine, the 2′deoxy version of 5-azacytidine, inhibits DNA methylation in a manner similar to 5-azacytidine but is not incorporated into RNA. *In vitro*, decitabine was shown to reverse the inactivation of *p16* in lung cancer cell lines by removing the transcriptional block of *p16* methylation (Merlo et al., 1995). Hypomethylating agents thus have sound rationale for use in the treatment of lung cancer. Unfortunately, clinical studies employing hypomethylating agents alone have been somewhat disappointing (Table 1).

**Table 1:** **Summary of selected epigenetic clinical trials in lung cancer**.

Treatment agents	Reference	Evaluable patients	Responses *n* (%)	SD, *n* (%)	TTP, (mos.)	OS, (mos.)
**HYPOMETHYLATING AGENT**						
Decitabine	Momparler et al. (1997)	9	0 (0)	4 (44)	N/R	6.7
Decitabine	Schrump et al. (2006)	16	0 (0)	3 (19)	N/R	N/R
**HDAC INHIBITOR**						
Entinostat	Ryan et al. (2005)	4	0 (0)	2 (50)	N/R	N/R
Vorinostat	Vansteenkiste et al. (2008)	8	0 (0)	6 (75)	1.2	N/R
Vorinostat	Traynor et al. (2009)	14	0 (0)	8 (57)	2.3	7.1
Romidepsin	Otterson et al. (2010)	16	0 (0)	3 (19)	1.8	6.0
**CHEMOTHERAPY COMBINATIONS**						
Vorinostat, carboplatin, paclitaxel	Ramalingam et al. (2010)	62	21 (34)	N/R	6.0	13.0
Entinostat, erlotinib	Witta et al. (2012)	67	2 (3)	N/R	1.97	8.9
**EPIGENETIC COMBINATIONS**						
Decitabine, valproic acid	Chu et al. (2013)	8	0 (0)	N/R	1.6	N/R
5-aza, sodium phenylbutyrate	Lin et al. (2009)	1	0 (0)	0 (0)	N/R	N/R
Hydralazine, magnesium valproate	Candelaria et al. (2007)	1	0 (0)	1 (100)	2.5	8.3
Decitabine, vorinostat	Stathis et al. (2011)	2	0 (0)	N/R	N/R	N/R
5-aza, entinostat	Juergens et al. (2011)	34	2 (6)	10 (29)	1.9	6.4

5-aza, 5-azacytidine; N/R, not reported; OS, overall survival; SD, stable disease; TTP, time to progression.

A phase I/II study of decitabine included 15 patients with untreated NSCLC, nine of whom were assessable (Momparler et al., 1997). Decitabine was administered as a continuous infusion over 8 h, repeated every 5–7 weeks. None of the patients with NSCLC had an objective response to therapy. Interestingly, four NSCLC patients had disease stabilization for at least 6 months and three patients had a survival of at least 15 months. Long-term follow-up of this study described one patient who survived for 81 months after treatment (Momparler and Ayoub, 2001). A phase I study of decitabine given as a continuous 72 h infusion every 34 days was later explored, seeking to maximize tumor suppressor gene expression in patients with thoracic malignancies (Schrump et al., 2006). While this study included a variety of tumors, there were 22 patients with lung cancer (19 with NSCLC and three with small cell lung cancer, SCLC), 16 of which were evaluable. Tolerability varied with the number of prior treatment regimens. Patients who had received two or fewer prior regimens had a maximum tolerated dose of 75 mg/m^2^ while those who had received three or more prior regimens had a maximum tolerated dose of 60 mg/m^2^. Again, there were no objective responses. Three patients with lung cancer had disease stabilization and for two patients, this control lasted for 10 months or more. Among patients who underwent repeated biopsies, one third displayed a molecular response with re-induction of *NY-ESO-1*, *MAGE-3*, or *p16* but again, none of these patients experienced a radiographic response to therapy. One reason why DNA methylation inhibitors alone may not be fully effective is that epigenetic control is complex and is mediated by many other molecular mechanisms, including the modification of histone tails.

## Histone Modifications

Two molecules each of histones 2A, 2B, 3, and 4 make up nucleosomes, around which much of the genome is wound. The N-terminal tails of the histones protrude from nucleosomes and post-translational modifications of these tails determine the accessibility of the DNA to transcription factors and other DNA-binding proteins (Strahl and Allis, 2000). One of the best-studied modifications, acetylation of lysine residues, reduces the tails’ positive charge and thereby their interaction with the negatively charged DNA backbone, relaxing the DNA (Clayton et al., 2006). This increases the availability of DNA to transcription factors and other regulatory proteins and generally increases expression. Conversely, deacetylation, mediated by HDACs generally leads to gene silencing. HDACs are frequently overexpressed in cancer cells, prompting deacetylation of histones and gene silencing. HDAC inhibitors, which restore the open conformation and tend to restore gene transcription, are highly promising anti-cancer therapeutics (Dokmanovic et al., 2007; Barneda-Zahonero and Parra, 2012).

In preclinical models, HDAC inhibition demonstrates promising antitumor activity. Several HDAC inhibitors (including LBH589, scriptaid, valproic acid, apicidin, OSU-HDAC-44, and MS-275) induce cell death in NSCLC cell lines (Brazelle et al., 2010; Tang et al., 2010). The phytochemical honokiol, an HDAC inhibitor, reduced the viability of several NSCLC cell lines, inducing a predictable G1 phase arrest, and inhibited the growth of lung tumor xenografts (Singh et al., 2013). Clinical experience (Table 1) includes a phase I study of the HDAC inhibitor entinostat in 31 patients with solid tumors that included four patients with NSCLC (Ryan et al., 2005). While no responses were seen, two of these patients had stabilization of their disease, one lasting 9 months. A multi-histology phase II study exploring three different doses included 10 patients with relapsed or refractory NSCLC (Vansteenkiste et al., 2008). Three patients received 200 mg twice daily, three received 300 mg twice daily and four received 400 mg twice daily. Two patients were not evaluable and in the remaining eight patients, there were no objective responses. The best response was stable disease for six patients and progressive disease for the other two. Another phase II study was conducted by the Wisconsin Oncology Network and included 16 patients with NSCLC (Traynor et al., 2009). This study explored a 400 mg daily dose of vorinostat given continuously in 21 day cycles. Two patients were inevaluable due to progressive disease during the first cycle. In the 14 evaluable patients, there were no objective responses. The best response was stable disease for eight patients. Interestingly, one patient had an uncharacteristically long duration of stable disease, with time to progression noted at 19.4 months.

Many studies, however, feature an HDAC inhibitor in combination with other treatments (Table 1). In preclinical models, HDAC inhibitors are synergistic with cytotoxic agents such as taxanes and platinum agents (Luchenko et al., 2011; Zuco et al., 2011). A phase II study in NSCLC of carboplatin and paclitaxel with randomization to the HDAC inhibitor vorinostat demonstrated a superior response rate (34.0 vs. 12.5%, *p* = 0.02) in the vorinostat arm with a trend toward an improvement in survival (Ramalingam et al., 2010). Combinations with targeted agents have been explored. In lung cancer cell lines resistant to EGFR tyrosine kinase inhibitors, treatment with trichostatin A, an HDAC inhibitor, can sensitize cells to therapy (Sharma et al., 2010). Clinically, however, the combination of entinostat and erlotinib was not superior to erlotinib alone in an unselected NSCLC population, though there were subsets of patients, including those with high E-cadherin expression, who may have derived greater benefit (Witta et al., 2012). Preclinical studies suggested that in NSCLC cell lines, one mechanism of resistance to vorinostat monotherapy is an increase in transcriptional activity of NF-kB involving the Akt pathway (Mayo et al., 2003). Inhibition of NF-kB *in vitro* sensitized NSCLC cells to apoptosis following exposure to HDAC inhibitors. This strategy was explored in a neoadjuvant phase I study combining vorinostat and bortezomib, a proteasome inhibitor (Jones et al., 2012). The maximum tolerated dose was established as bortezomib 1.3 mg/m^2^ weekly and vorinostat 300 mg twice daily. While no radiographic responses were noted in this relatively brief window-of-opportunity study, encouraging correlates were described including necrosis in 30% of tumors and a decrease in serum 20S proteasome activity in 50% of patients. In addition, comparison of gene expression arrays from samples before and after treatment detected unique patterns of upregulation and downregulation that may provide mechanistic insight into response and potentially guide therapeutic use. Future studies are under consideration.

## Combination Epigenetic Therapy

Given their distinct roles in epigenetic control, there is rationale to combine inhibitors of DNA demethylation and histone deacetylation to potentiate gene reactivation. This approach has demonstrated promising synergy in preclinical models. Hypermethylation silences *MLH1*, *TIMP3*, and *CDKN2A* in colorectal cancer cells. Treatment with either the HDAC inhibitor trichostatin A or the hypomethylating agent decitabine alone does not restore transcription of these genes (Cameron et al., 1999) but the combination of these two agents reversed silencing and led to significant re-expression. In lung cancer cell lines, the HDAC inhibitor depsipeptide acetylates histones H3 and H4 (Zhu et al., 2001) but this effect is significantly magnified when cells are pretreated with decitabine. The combination of the DNMT inhibitor 5-azacytidine and the HDAC inhibitor entinostat inhibited growth of *K-ras/p53* mutant lung adenocarcinoma in an orthotopic lung cancer mouse model (Belinsky et al., 2011). Epigenetic analysis demonstrated demethylation across hundreds of genes and re-expression of several critical genes including *p16*.

These preclinical data prompted several trials employing this strategy (Table 1). A phase I study combined decitabine and valproic acid in eight patients with NSCLC (Chu et al., 2013). No responses were seen with this combination. However, dosing and compliance were challenging due to neurotoxicity attributed to valproic acid. A phase I study of 5-azacytidine and the HDAC inhibitor sodium phenylbutyrate was much better tolerated, though the sole patient with lung cancer did not respond to therapy (Lin et al., 2009). A phase II study of the weak DNA hypomethylating agent hydralazine and the HDAC inhibitor magnesium valproate was also well tolerated (Candelaria et al., 2007). While there were patients who experienced a response with this combination, the sole patient with lung cancer only achieved transient stable disease lasting 2.5 months. A subsequent phase I study assessed the combination of decitabine and vorinostat (Stathis et al., 2011). Decitabine was given intravenously for five consecutive days and vorinostat was administered orally in a sequential manner starting on day 6 or in a concurrent manner starting on day 3. Cycles were repeated every 28 days. The maximum tolerated dose of these agents was the same in both arms: decitabine was 10 mg/m^2^ and vorinostat was 200 mg twice daily. The sequential schedule was better tolerated and recommended for further study; however, no patients responded to therapy, including the two patients with NSCLC.

The combination of 5-azacytidine and entinostat has shown more promise. A phase I/II study combined these two agents in patients with progressive and metastatic NSCLC (Juergens et al., 2011). There were no dose limiting toxicities at the planned dose levels, which were well below the maximum tolerated doses for each drug. Of the 34 evaluable patients who participated, 10 had stable disease lasting at least 12 weeks. One patient had a partial response lasting 8 months and another patient had a durable, complete response lasting 14 months. There has been minimal toxicity with this combination and an expansion cohort to further define activity is being planned (NCT00387465). Interestingly, the clinical responses persisted beyond cessation of epigenetic therapy and many participating patients achieved notable responses to subsequent salvage therapy. In this study, 19 patients received subsequent systemic therapy within 6 months and four of these patients (21%) achieved major objective responses. This has led to the development of several trials incorporating sequential therapy that starts with epigenetic “priming.” One planned trial randomizes patients with pretreated NSCLC to standard cytotoxic chemotherapy alone or following treatment with 5-azacytidine and entinostat. A separate single arm phase II study in development for NSCLC explores the efficacy of nivolumab (BMS-936558), a PD-1 monoclonal antibody, after two cycles of 5-azacytidine and entinostat epigenetic therapy.

The combination of 5-azacytidine and entinostat is also being explored in the adjuvant setting. An ongoing trial is examining the role of this combination in patients with resected stage I NSCLC (NCT01207726). Standard of care is observation, after several analyses demonstrated a lack of benefit to cytotoxic chemotherapy in this population, despite a 5-year survival of only 60% (Pignon et al., 2008). There is reason to implicate epigenetic deregulation in recurrence of resected NSCLC. Methylation signatures are associated with the development of lung cancer among patients at high risk (Belinsky et al., 2006) and the risk of recurrence after resection of stage I NSCLC (Brock et al., 2008). This provides the rationale for the use of epigenetic therapy in this patient population. In this trial, patients are randomized to observation or treatment with 5-azacytidine and entinostat for six cycles. The primary objective is to assess the impact of this treatment on 3 year progression-free survival.

The examples above illustrate the promise of epigenetic therapy and its active investigation. Data to date suggest that it is the gene reactivation properties that underlie the therapeutic efficacy of these drugs. However, evidence for epigenetic activation of tumor promoting genes has been reported (Selamat et al., 2012), pointing to the need for a deeper understanding of epigenetic regulation and the consequences of epigenetic therapies. This is very well illustrated by the complexities of gene regulation by miRNAs.

## MicroRNAs

MicroRNAs are small non-coding RNAs that regulate gene expression at the post-transcriptional level (Ambros, 2003; Chen et al., 2012). A single miRNA can target many genes and depending on whether it targets proto-oncogenes or tumor suppressor genes, its expression may be up or down-regulated in cancer (Croce, 2009). MiRNA-encoding genes can undergo the same epigenetic (de)regulation of any other protein-coding gene, such as promoter methylation, histone modifications, and chromatin changes (Fabbri and Calin, 2010; Baer et al., 2013). In lung cancer, the let-7a-3 promoter was found to be hypomethylated in lung adenocarcinoma primary tumors compared to normal tissue, suggesting an oncogenic role for this miRNA in lung cancer (Brueckner et al., 2007). In contrast, miR-9-3 and miR-193a are silenced by DNA methylation in NSCLC and the presence of methylated miR-9-3 was a poor prognostic factor predicting shorter overall survival (Heller et al., 2012). MiRNAs can also be silenced by histone modifications. For instance, increased levels of repressive H3K27 trimethylation and H3K9 dimethylation marks were seen on the miR-212 promoter in lung cancer cell line Calu-1, which expresses low levels of miR-212, compared to a human fetal lung fibroblast cell line showing high miR-212 expression, and the effect of epigenetic modifying drugs such as HDACs supported a role for histone modifications in miR-212 regulation (Incoronato et al., 2010; Incoronato et al., 2011).

While epigenetic effects can control miRNA expression, the reverse is also true: DNMTs can be regulated by miRNAs in lung cancer cells (Fabbri et al., 2007, 2013). Specifically, the miR-29 family (composed of 29a, 29b, and 29c) was shown to bind directly to the 3′UTR region of DNMT3A and 3B (*de novo* methyltransferases), two key enzymes involved in DNA methylation. Members of the miR-29 family members are frequently down-regulated in NSCLC compared to adjacent non-tumor lung, and their expression is inversely correlated with the expression levels of DNMT3A and 3B (Fabbri et al., 2007). Restoration of miR-29 down-regulated DNMT3A and DNMT3B, inducing a global hypomethylated state in cancer cells and concomitant re-expression of tumor suppressor genes such as *FHIT* and *WWOX*, whose expression is silenced in NSCLC by promoter hypermethylation. More recently, it was shown that in addition to directly targeting DNMT3A and 3B, miR-29b also indirectly targets DNMT1 by silencing its transactivator SP1, in acute myeloid leukemia (Garzon et al., 2009). MiR-29s thus represents the prototype of an “epi-miRNA”: a miRNA that targets effectors of the epigenetic machinery.

In addition to targeting DNMTs, epi-miRNAs can also target HDACs (Chen et al., 2006; Tuddenham et al., 2006) and Polycomb Group Proteins (PcG), which mediate gene silencing and can promote the survival and metastasis of cancer cells (Varambally et al., 2008). A constant flood of new studies reports the existence of additional epi-miRNAs, the identification of new roles for already identified epi-miRNAs and new layers of epigenetic regulation of miRNAs. These studies illustrate the complex interplay between miRNAs and epigenetics, pointing to the importance of understanding these relationships to properly design cancer therapies targeting miRNAs and epigenetics (Croce, 2011).

Based on the importance of miRNAs in cancer and their ability to influence gene expression, they are under investigation as therapeutic tools that can potentially target any RNA of interest. MiRNAs can either be delivered directly to cancer cells as synthetically generated miRNA-mimic molecules or can be administered as anti-miRNA molecules if the miRNA needs to be silenced. Most commonly, anti-miRNAs are administered as antagomirs (Krutzfeldt et al., 2005) or locked-nucleic acid (LNA) anti-miRNAs (Castoldi et al., 2006), which are oligonucleotides complementary to the sequence of the targeted mature miRNA, but biochemically modified to reduce the risk of degradation by cellular RNAses and conjugated with cholesterol to facilitate entry into cells. While miRNA-mimics and anti-miRNAs affect miRNA expression and therefore the expression of any mRNAs targeted by them, another approach is to silence specific genes. To this end, both small-interfering RNAs (siRNAs) and genetically encoded expression vectors encoding small hairpin RNAs (shRNAs) that are processed by the miRNA machinery have been used (Rao et al., 2009). This approach involves post-transcriptional, sequence-specific silencing of genes critical to the survival and proliferation of lung cancer cells and has shown encouraging *in vitro* activity in some cancer models. Silencing specific genes with siRNA can impact relevant signaling pathways, such as VEGF for angiogenesis (He et al., 2008) or survivin for growth and invasion (Kunze et al., 2008). This strategy has been associated with antitumor activity *in vitro* (Odate et al., 2013), though the optimal target is unclear (Tong, 2006). RNA interference may be better suited to enhance cytotoxic agents. STAT3 targeted siRNA sensitized lung cancer cells to cisplatin and doxorubicin (Kulesza et al., 2013) while PLK1 targeted shRNA provided an additive inhibition of lung adenocarcinoma cell survival when administered with gemcitabine (Zhou et al., 2012). Many studies demonstrate similar effects with other agents (Zhou et al., 2012; Zou et al., 2013). Among the challenges of translating preclinical findings to clinical studies is the relatively inefficient delivery of siRNA to target cells, in part due to poor uptake and short half-life (Chen and Zhaori, 2010). This can be alleviated somewhat by using lentiviral delivery of genes encoding shRNAs which can then be stably expressed long-term (Rao et al., 2009). However, infecting all cancer cells will remain challenging. While these strategies have shown potential in the treatment of lung cancer, clinical experience is quite limited to date.

## Small Cell Lung Cancer

Most of the preclinical and clinical experience in lung cancer with epigenetic therapy has been focused on NSCLC, perhaps due to the fact that our understanding of epigenetic deregulation in SCLC is in its infancy (Kalari et al., 2012). This is likely due to the limited availability of clinical samples from SCLC patients. Nevertheless, some studies in SCLC have been conducted. A phase II study of the HDAC inhibitor romidepsin in patients with relapsed chemosensitive SCLC did not demonstrate significant clinical activity (Otterson et al., 2010). There were no responses in 16 patients and the median progression-free survival was only 1.8 months, though three patients (19%) did achieve stable disease. Just as in NSCLC, combination therapy is felt to have more promise than monotherapy in SCLC. *In vitro*, combining DNMT inhibition with HDAC inhibition was found to have a greater pro-apoptotic effect than monotherapy (Kaminskyy et al., 2011). In similar *in vitro* models, decitabine showed a synergistic induction of DNA damage in the context of HDAC inhibition (Luszczek et al., 2010). The synergy between HDAC inhibition and various cytotoxic agents has also been demonstrated in preclinical SCLC models (Luchenko et al., 2011). As our understanding of the epigenetic underpinnings of SCLC development and progression increases, the development and application of epigenetic therapies are sure to follow.

## Concluding Remarks

The prevalence of epigenetic modifications in lung cancer and their role in carcinogenesis underscore the great potential of epigenetic therapeutic strategies. Early clinical trials, however, have yielded underwhelming responses to epigenetic agents. Studies featuring monotherapy have failed to document a single response and the most promising combination of epigenetic agents demonstrated a response rate of only 6%. Yet it is clear that there are patients deriving benefit from therapy, with some patients achieving uncharacteristically long periods of disease control and survival. The few responses seen with combination therapy have been both extensive and durable. Perhaps radiographic response as measured by standard RECIST criteria is not the best means of establishing efficacy for epigenetic therapy. Epigenetic agents can induce differentiation, potentially by inducing re-expression of tumor suppressor genes, and these differentiated cells are then prone to cell death. This process may be much slower than apoptosis induced by traditional cytotoxic agents and thus, less readily apparent on radiographic studies. The interpretation and definition of response to epigenetic therapy must evolve as we gain more experience. Clinically, time to progression may be a more reflective endpoint to gage efficacy in this class of agents. In addition, the optimal use of epigenetic therapy may be in conjunction with cytotoxic agents, based on the frequency of responses to subsequent therapies. Studies exploring this epigenetic “priming” strategy are already being designed.

As new trials open to accrual, it will be important to identify predictive markers to guide the use of epigenetic therapy. Our understanding of how and why these agents work remains rudimentary and must be expanded. Pharmacodynamic changes in gene expression (Jones et al., 2012) will offer insight into the downstream effects of these agents and could provide useful predictive markers for epigenetic therapies. The development of novel molecular markers to monitor epigenetic responses, such as assessment of DNA methylation in bodily fluids (Juergens et al., 2011) may also provide a measure of molecular response to the drugs, and will be a key element to incorporate in future studies. Further investigation of the epigenomic profiles of various epigenetic effectors, including DNA methylation, histone marks, chromatin conformation, and miRNAs will be of great importance for the development of new targeted therapies. In addition, a better appreciation of the interaction between epigenetic and genetic alterations will be crucial moving forward. Such studies will likely reveal additional subtypes of lung cancer that respond to unique treatment strategies, allowing refinement of treatment paradigms and tailoring to unique (epi)genetically distinct subsets of lung cancer.

## Conflict of Interest Statement

The authors declare that the research was conducted in the absence of any commercial or financial relationships that could be construed as a potential conflict of interest.
